# Simulating therapeutic drug monitoring results for dose individualisation to maintain investigator blinding in a randomised controlled trial

**DOI:** 10.1186/s13063-017-1992-6

**Published:** 2017-06-07

**Authors:** Maia Lesosky, John Joska, Eric Decloedt

**Affiliations:** 1 Division of Epidemiology and Biostatistics, School of Public Health and Family Medicine, University of Cape Town, Cape Town, Western Cape South Africa; 2 Division of Neuropsychiatry, Department of Psychiatry and Mental Health, Faculty of Health Sciences, University of Cape Town, Cape Town, Western Cape South Africa; 3 Division of Clinical Pharmacology, Department of Medicine, Faculty of Medicine and Health Sciences, Stellenbosch University, Stellenbosch, Western Cape South Africa

**Keywords:** Dose adjustment, Blinding, Simulation

## Abstract

**Background:**

Therapeutic drug monitoring (TDM) is essential practice when dosing drugs with a narrow therapeutic index in order to achieve a plasma drug concentration within a narrow target range above the efficacy concentration but below the toxicity concentration. However, TDM with dose individualisation is challenging during a double-blind clinical trial with laboratory staff and investigators blinded to treatment arm allocation.

**Methods:**

Drug concentrations were simulated for participants in the placebo arm by an unblinded independent statistician, utilising the measured values from the treatment arm participants. Simulated and actual concentrations were re-blinded and passed on to a dose-adjusting investigator, who made dose adjustment recommendations but was not directly responsible for clinical care of participants.

**Results:**

A total of 257 sham lithium plasma concentrations were simulated utilising 242 true lithium plasma concentrations in real time as the trial progressed. The simulated values had a median (interquartile range) of 0.59 (0.46, 0.72) compared to 0.53 (0.39, 0.72) in the treatment arm. Blinding of the laboratory staff and dose-adjusting investigator was maintained successfully.

**Conclusions:**

We succeeded in simulating sham lithium plasma concentrations while maintaining blinding. Our simulated values have a smaller range than the observed data, which can be explained by the challenges with respect to drug adherence and dose timing that were experienced.

**Trial registration:**

Pan African Clinical Trials Registry, PACTR201310000635418. Registered on 30 August 2013.

## Background

Therapeutic drug monitoring (TDM) is essential when dosing drugs with a narrow therapeutic index to achieve a plasma drug concentration within a narrow target range that is above the efficacy concentration but below the toxicity concentration. However, TDM with dose individualisation is challenging during a double-blind clinical trial with both laboratory staff and investigators blinded to treatment arm allocation. We report our experience of simulating TDM results for dose individualisation by an investigator while maintaining blinding in a double-blind, placebo-controlled, randomised trial (RCT). The trial evaluated the 24-week efficacy and safety of lithium in patients with moderate to severe HIV-associated neurocognitive disorder (HAND). The trial was conducted in response to the rising overall prevalence of HAND [1, 2]. Preliminary data suggested that lithium may provide clinical benefit as an adjunctive treatment in patients established on ART [3]. Lithium has a narrow therapeutic index (0.6–1.0 mmol/L), and the study team had to perform lithium TDM and individualise dosing in conjunction with participant adverse events, while maintaining investigator blinding [4].

## Methods

The RCT methods, patient population, and primary outcomes are described elsewhere [5], but briefly, this was a 24-week, two-arm, placebo-controlled phase IIb trial to study lithium as an adjunctive pharmacotherapy in individuals with moderate to severe HAND. Participants were individually randomised to either a placebo or lithium arm using block randomisation prior to the start of the study. Lithium plasma concentrations were measured in all participants irrespective of treatment allocation at scheduled study visits.

### Blinding process

The laboratory reported all concentrations directly to the study statistician; placebo concentrations were reported as lower than the limit of detection. The study statistician simulated sham plasma drug concentrations, as detailed below, and provided re-blinded results to the dose-adjusting investigator, who individualised lithium dosing in conjunction with adverse event reports containing causality assessment and severity grading. Lithium was titrated to achieve a target plasma concentration between 0.6 and 1.0 mmol/L, aiming for 20 mg/kg/day, as per study protocol.

The dose-adjusting investigator was not directly involved in participant care and received adverse event reports from other investigators. Dosing was adjusted in order to achieve target serum concentrations assuming linear lithium pharmacokinetics. When significant adverse events were considered to be related to the study drug, doses were not upward adjusted, even when concentrations were below 0.6 mmol/L. When concentrations were below 0.6 mmol/L at usual therapeutic doses of lithium (regarded to be between 500 to 1500 mg daily) and upward adjustment was felt to be unsafe, a note was sent to the investigators to confirm adherence without dose adjusting.

The study investigators who were involved in participant care, the laboratory staff, and the dose-adjusting investigator (who was not involved in participant care) remained blinded until study close. The statistician was not blinded, as unblinding was required in order to carry out statistical procedures. The statistician communicated with the dose-adjusting investigator through template emails and never communicated with the blinded study investigators.

### Statistical methods

There was minimal data available prior to trial initiation on normal or reference ranges for plasma lithium concentrations in this population, so the decision was made to use trial-collected data in real time to set model parameters. Mean and standard deviation were estimated from available plasma lithium concentrations measured in the treatment arm and sham values sampled from a Gaussian distribution based on those estimates. Rejection sampling was used to ensure simulated values were inside the therapeutic range of 0.6–1.0 mmol/L. The 0.6 mmol/L threshold was modified in consultation with the Data Safety and Monitoring Board to a lower threshold of 0.3 mmol/L after poor adherence in the treatment arm as well as lower than expected concentrations became apparent. Plasma lithium concentrations were reported in a single file with no treatment arm information and included the concentrations as received for the treatment arm (including those below the assay limit of detection) and the sham values for the placebo arm.

The study was approved by the human research ethics committees of the University of Cape Town (071/2013) and Stellenbosch University (M13/07/027) and registered on the Pan African Clinical Trials Registry (PACTR201310000635418).

## Results

**Fig. 1:**
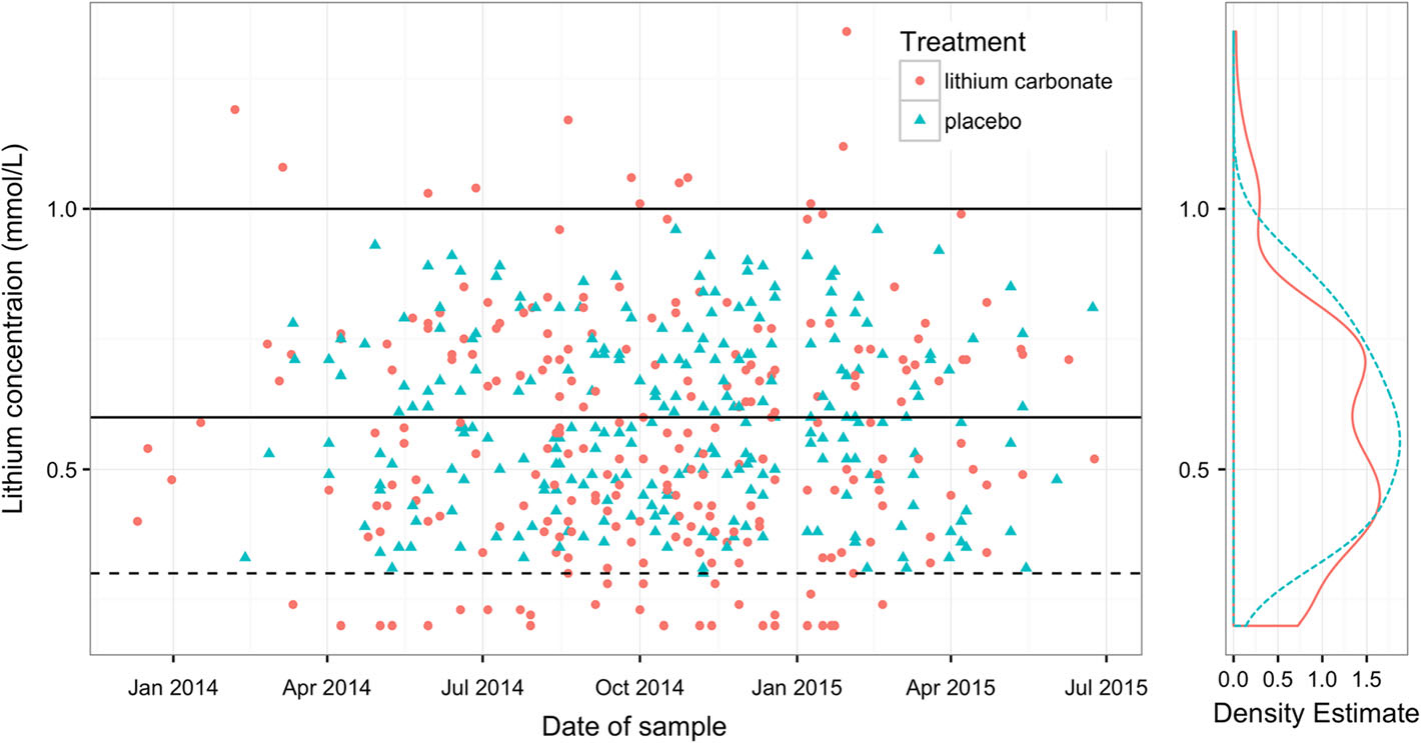
Observed plasma lithium concentrations in individuals randomised to treatment arm (*orange circles*) and sham lithium concentrations simulated for individuals randomised to placebo arm (*blue triangles*) by date of sample. *Solid horizontal lines* represent therapeutic range; *dashed horizontal line* represents adjusted lower threshold. Distribution of simulated and measured lithium concentration density estimates provided as marginal plot (*dashed* = placebo; *solid* = lithium)

A total of *n* = 257 sham lithium plasma concentrations were simulated, with an overall median (interquartile range, IQR) of 0.59 (0.46, 0.72) mmol/L compared to the *n* = 242 observed measurements in the treatment arm with median (IQR) 0.53 (0.39, 0.72) mmol/L. The scatter plot and density estimates by arm are given in Fig. 1. The dose-adjusting investigator made dose-change recommendations to increase or decrease the dose on a total of 182 occasions (55% (*n* = 100) in the treatment arm vs 45% (*n* = 82) in the placebo arm). Dose decreases were recommended 33 times (54% (*n* = 18) vs 45% (*n* = 15)) and dose increases recommended 149 times (54% (*n* = 82) vs 45% (*n* = 67)) in the treatment and placebo arms respectively. Adverse events and poor adherence records, as reported in [5], were evenly distributed by treatment arm.

## Discussion

We succeeded in simulating sham lithium plasma concentrations while maintaining blinding, as evidenced by similar rates of dose adjustments in each arm. Our simulated values have a smaller range than the observed data, which can be explained by the challenges that we encountered. Early adjustment of the lower bound of the simulated range to account for non-adherence in the treatment arm was required to prevent inadvertent unblinding of the dose-adjusting investigator. However, the implications of simulating data that would initiate an intervention (in this case counselling regarding adherence) for participants who may have been fully adherent prevented adjustment to the full range of observed concentrations. The observed concentration range can be explained by both low adherence and sub-optimal dose timing. Ideally, lithium TDM should be performed as a trough concentration just prior to the next dose being given, but this was not always the case due to participants not always arriving on time for their appointments. Our simulation procedure has a number of limitations. First, we did not generate sham adverse event reports, as we reasoned that dose individualisation in response to sham adverse event report may potentially jeopardise efficacy and increase risk. Second, it would have been ideal to simulate individual time trajectories instead of independently simulated values at each time point. However, with sparse data available, this was not feasible. In settings where the data to be simulated is better described, this may be possible and desirable. 

Trial processes and procedures must be adjusted to take real-time feedback into account, and in particular, this process required constant availability by the statistician. In practice, especially with trials of longer duration, multiple persons must be identified who can carry out the procedure in the event of absence of the primary statistician. The other possibility is to develop a semi-automated procedure by using some of the new programming tools, for example, the ‘Shiny’ package in R [6], which allows for interactive interfaces to R software via a website.

The balance between maintaining blinding in RCTs and patient safety must always err towards patient safety, and we have demonstrated a feasible method of maintaining investigator blinding while allowing for TDM and dose individualisation. Rapid response and willingness to modify protocol must be considered in light of trial data in order to maintain effective blinding.

## Conclusions

The balance between maintaining blinding in RCTs and patient safety must always err towards patient safety, and we have demonstrated a feasible method of maintaining investigator blinding while allowing for TDM and dose individualisation. Rapid response and willingness to modify protocol must be considered in light of trial data in order to maintain effective blinding.
